# BMAL1 functions as a cAMP-responsive coactivator of HDAC5 to regulate hepatic gluconeogenesis

**DOI:** 10.1007/s13238-018-0514-y

**Published:** 2018-03-05

**Authors:** Jian Li, Sihan Lv, Xinchen Qiu, Jiamin Yu, Junkun Jiang, Yalan Jin, Wenxuan Guo, Ruowei Zhao, Zhen-Ning Zhang, Chao Zhang, Bing Luan

**Affiliations:** 1 Department of Endocrinology, Shanghai Tenth People’s Hospital, School of Medicine, Tongji University, Shanghai, China; 2 Translational Medical Center for Stem Cell Therapy & Institute for Regenerative Medicine, Shanghai East Hospital, Shanghai Key Laboratory of Signaling and Disease Research, School of Life Sciences and Technology, Tongji University, Shanghai, China

**Dear Editor,**

Gluconeogenesis is one of the major mechanisms to maintain hepatic glucose homeostasis and dysregulation of hepatic gluconeogenesis contributes to hyperglycemia in type 2 diabetes. Under fasted conditions, increases in circulating glucagon promote hepatic glucose production through activation of gluconeogenic pathway by HDAC5 and CREB coactivator CRTC2 (Lv et al., 2016; Lv et al., 2017; Qiu et al., 2017). The circadian clock coordinates behavior and metabolism into rhythms not only in the central hypothalamus but also in peripheral tissues (Marcheva et al., 2010; Vollmers et al., 2009). Transcription factor BMAL1 heterodimerizes with CLOCK to activate the expression of *Per* and *Cry*, which in turn suppress CLOCK/BMAL1 activity (Reppert & Weaver, 2002). Although BMAL1 knockout mice show fasting hypoglycemia (Rudic et al., 2004), the detailed mechanism of BMAL1 regulation on hepatic gluconeogenesis has not been thoroughly understood.

To explore the function of BMAL1 on hepatic gluconeogenesis, we generated liver-specific BMAL1 knockout mice (BMAL1 LKO) by crossing BMAL1 flox/flox mice with Albumin-Cre transgenic mice. BMAL1 LKO mice were indistinguishable from their wild-type littermates in body weight and blood glucose levels under ad lib and fasted conditions (Fig. S1A and S1B). However, fasting serum insulin levels were relatively lower in BMAL1 LKO mice compared with WT littermates (Fig. S1C). When challenged with sodium pyruvate, BMAL1 LKO mice showed decreased gluconeogenesis as assayed by pyruvate tolerance test (PTT) (Fig. S1D). Similarly, the expression of endogenous gluconeogenic genes including *G6Pase*, *Pck1* and *Pgc1a* was decreased in the liver (Fig. S1E), suggesting a pro-gluconeogenic effect of BMAL1. Supporting this scenario, adenovirus-mediated BMAL1 expression (Ad-BMAL1) in primary hepatocytes greatly prompted forskolin (FSK)-induced gluconeogenic gene expression (*G6Pase*, *Pck1*), while BMAL1 knockdown mediated by adenovirus encoding BMAL1 RNAi (Ad-BMAL1i) inhibited gluconeogenic gene expression (Figs. 1A, 1B, S1F and S1G).

**Figure 1:**
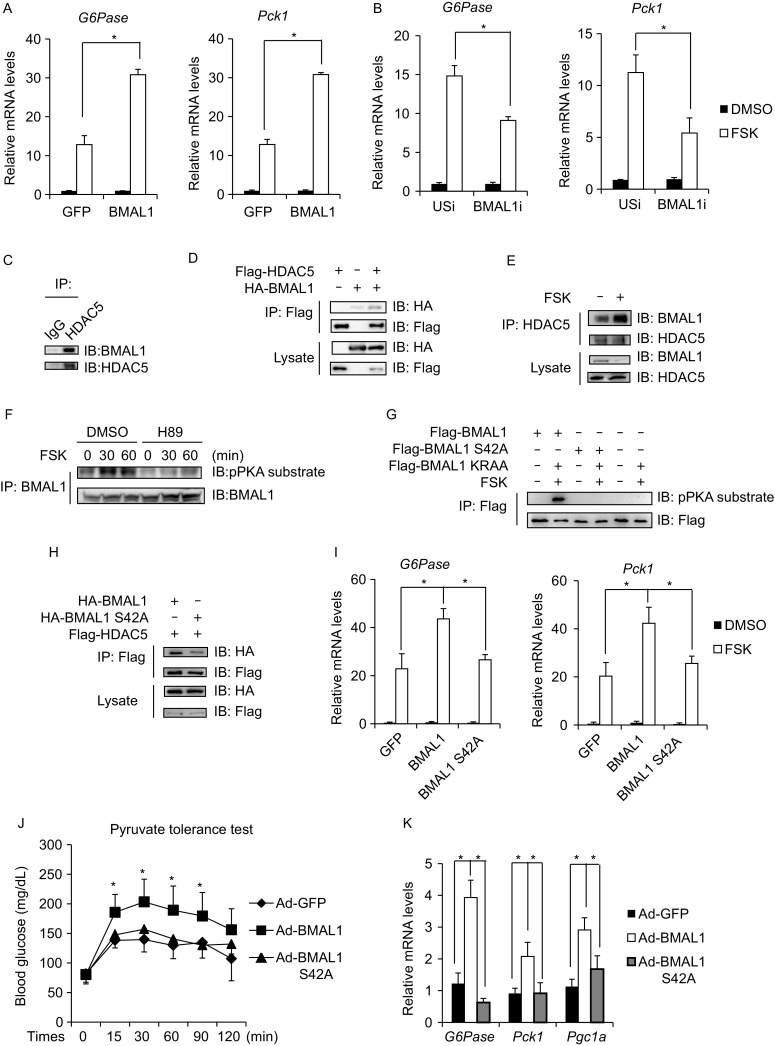
**BMAL1 promotes hepatic gluconeogenesis via phosphorylation-dependent interaction with HDAC5**. (A) Effect of Ad-BMAL1 on FSK-induced *G6pase* and *Pck1* mRNA amounts in primary hepatocytes (*n* = 3). (B) Effect of Ad-BMAL1i on FSK-induced *G6pase* and *Pck1* mRNA amounts in primary hepatocytes (*n* = 3). (C) Immunoblot of BMAL1 recovered from immunoprecipitations of HDAC5 from primary hepatocytes. (D) Interaction between Flag-tagged HDAC5 and HA-tagged BMAL1 in HEK293T cells. (E) Immunoblot of BMAL1 recovered from immunoprecipitations of HDAC5 from primary hepatocytes exposed to FSK. (F) Immunoblot showing effects of H89 treatment on FSK-induced BMAL1 phosphorylation at indicated time in primary hepatocytes. (G) Immunoblot showing effects of FSK treatment on BMAL1 phosphorylation in HEK293T transfected with indicated constructs. (H) Interaction between Flag-tagged HDAC5 and HA-tagged BMAL1 or BMAL1 S42A in HEK293T cells. (I) Effect of Ad-BMAL1 or Ad-BMAL1 S42A on FSK-induced *G6Pase* and *Pck1* mRNA amounts in primary hepatocytes (*n* = 3). (J) Pyruvate tolerance test in mice injected with Ad-BMAL1 or Ad-BMAL1 S42A (*n* = 6). (K) Effect of fasting on mRNA amounts for hepatic gluconeogenic genes in liver of mice injected with Ad-BMAL1 or Ad-BMAL1 S42A (*n* = 6). All data are presented as mean ± s.e.m. *P < 0.05

Since hepatic gluconeogenic gene expression is mainly regulated by the CREB/CRTC2 pathway and HDAC5/FOXO1 pathway (Altarejos & Montminy, 2011; Mihaylova et al., 2011; Wang et al., 2011), we tried to identify which pathway contributes to the effect of BMAL1 on gluconeogenic gene expression. The promoter of G6Pase, a rate-limiting enzyme for gluconeogenesis, contains binding sites of both CREB/CRTC2 (cAMP response element, CRE) and HDAC5/FOXO1 (insulin response element, IRE) (Lan et al., 2007; Liu et al., 2008). Similar to the effect on endogenous *G6Pase* mRNA expression, Ad-BMAL1 expression increased FSK-induced *G6Pase*-luc activity in primary hepatocytes (Fig. S2A). In contrast, FSK-induced CRE-luc (composed of 3 tandem copies of CRE but not IRE sites) activity was not influenced by Ad-BMAL1 expression (Fig. S2B). Furthermore, the effect of Ad-BMAL1 expression on *G6Pase*-luc activity was largely abolished when IRE sites but not CRE sites were mutated, suggesting that BMAL1 specifically regulates HDAC5 pathway (Fig. S2C). Consistent with the results of Ad-BMAL1 expression, knockdown of BMAL1 by Ad-BMAL1i resulted in attenuation of *G6Pase*-luc activity in primary hepatocytes (Fig. S2D). HDAC5 is phosphorylated by SIK kinases and sequestered in the cytoplasm under basal conditions. Exposure to FSK triggers HDAC5 dephosphorylation and nuclear translocation where they associate with the promoters of gluconeogenic enzymes such as G6Pase (Mihaylova et al., 2011). Based on the ability of BMAL1 to promote HDAC5-mediated gluconeogenic gene expression, we tested whether BMAL1 modulated HDAC5 dephosphorylation upon FSK stimulation. Indeed, Ad-BMAL1 expression in primary hepatocytes greatly enhanced FSK-induced HDAC5 dephosphorylation, while BMAL1 knockdown by Ad-BMAL1i blocked HDAC5 dephosphorylation upon FSK stimulation (Fig. S2E and S2F). In contrast, FSK-induced CREB phosphorylation was not influenced by BMAL1 overexpression or knockdown (Fig. S2E and S2F). Collectively, these data show that BMAL1 promotes gluconeogenic gene expression through HDAC5 pathway.

We next tested whether BMAL1 associates with HDAC5. Supporting this idea, we recovered endogenous BMAL1 from immunoprecipitations (IPs) of endogenous HDAC5 in primary hepatocytes (Fig. 1C). We obtained similar results in co-IP studies of HEK293T cells transfected with epitope-tagged BMAL1 and HDAC5 expression vectors (Fig. 1D). Interestingly, exposure to FSK greatly increased the endogenous BMAL1 and HDAC5 interaction in primary hepatocytes (Fig. 1E). Consistent with the co-IP studies, DsRed-tagged HDAC5 shuttled to nucleus upon FSK treatment, where they colocalized with GFP-tagged BMAL1 in HEK293T cells (Fig. S3A). Taking into consideration the important function of PKA on gluconeogenic gene expression, we speculated that FSK might induce PKA-mediated phosphorylation of BMAL1. Indeed, exposure of primary hepatocytes to FSK induced the phosphorylation of BMAL1 in a time dependent manner as evidenced by immunoblot with anti-phospho-PKA substrate antibody (Fig. S3B); these effects were blocked by H89 (PKA inhibitor) (Fig. 1F). Consistently, phosphorylation of BMAL1 was increased in the liver of fasted mice in comparison with ad lib mice (Fig. S3C). PKA specifically phosphorylates substrates at a consensus serine/threonine residue with arginine at the –3 position (Montminy, 1997; Pearson & Kemp, 1991). BMAL1 contains 7 conserved PKA phosphorylation sites (Ser42, Thr129, Ser241, Ser321, Ser336, Ser422 and Ser513) and mutations of each putative site were tested to identify the specific site of PKA phosphorylation on BMAL1. As a result, Ser42 to Ala (S42A) mutation but not others completely disrupted BMAL1 phosphorylation by FSK stimulation (Figs. 1G, S3D and S3E). Furthermore, mutation of Lys38 and Arg39 to Ala (KRAA) which disrupted the PKA consensus motif also led to the loss of PKA phosphorylation on BMAL1 (Fig. 1G). The PKA-mediated phosphorylation of BMAL1 at Ser42 prompted us to explore its contribution to FSK-induced BMAL1-HDAC5 interaction. BMAL1 S42A as well as KRAA mutations interacted with HDAC5 with lower affinity compared with wild type BMAL1 (Figs. 1H and S3F). PKA-mediated dephosphorylation of HDAC5 also contributed to the BMAL1-HDAC5 interaction, as phosphorylation-defective HDAC5 mutant (HDAC5 S259/498A, HDAC5 2SA) showed higher affinity to interact with BMAL1 (Fig. S3G). Collectively, these results indicate that FSK stimulates BMAL1-HDAC5 interaction via PKA-mediated BMAL1 phosphorylation.

To further evaluate the role of BMAL1-HDAC5 interaction on hepatic gluconeogenesis, primary hepatocytes were infected with adenovirus encoding either BMAL1 (Ad-BMAL1) or BMAL1 S42A (Ad-BMAL1 S42A). FSK induced *G6Pase*-luc activity as well as gluconeogenic gene expression (*G6Pase*, *Pck1*) was enhanced by Ad-BMAL1, however, no such effect was observed for Ad-BMAL1 S42A expression (Figs. S3H and S1I). Furthermore, while FSK-induced HDAC5 dephosphorylation was dramatically promoted by Ad-BMAL1 expression, Ad-BMAL1 S42A failed to show any effect (Fig. S3I). Based on these results, we further evaluated the importance of BMAL1-HDAC5 interaction on the gluconeogenesis *in vivo*. Mice were injected intravenously with Ad-GFP, Ad-BMAL1 or Ad-BMAL1 S42A. Although blood glucose levels under ad lib and fasted conditions were not dramatically influenced (Fig. S3J), the hepatic gluconeogenesis as assessed by PTT as well as hepatic gluconeogenic gene expression was increased in Ad-BMAL1 injected mice but not in Ad-BMAL1 S42A injected mice (Fig. 1J and 1K).

Given that HDAC5/FOXO1-dependent increase in gluconeogenesis is a common feature of insulin resistance and obesity (Gross et al., 2009), we wondered whether unregulated hepatic BMAL1-HDAC5 interaction might contribute to increases in gluconeogenesis in this setting. Supporting this notion, hepatic amounts of PKA-phosphorylated BMAL1 were significantly increased in HFD-fed mice compared with RD-fed mice (Fig. 2A), suggesting an increased interaction. Indeed, binding affinity of BMAL1 with HDAC5 was dramatically increased in liver of HFD-fed mice compared with RD-fed mice (Fig. 2B). Changes in BMAL1 phosphorylation and BMAL1-HDAC5 interaction in HFD-fed mice indicate their pathophysiological role under diabetic conditions and prompt us to further investigate its contribution to obesity-associated hyperglycemia using BMAL1 LKO mice upon HFD feeding. Indeed, although body weight was not significantly altered, BMAL1 LKO mice showed decreased fasting blood glucose levels, hepatic gluconeogenesis as assessed by PTT, as well as hepatic gluconeogenic gene expression in comparison with littermates under fasted stage (Fig. 2C–F). These results suggest that inhibition of hepatic BMAL1 could be a new therapeutic approach to ameliorate hyperglycemia by suppressing gluconeogenesis in diabetes.

**Figure 2:**
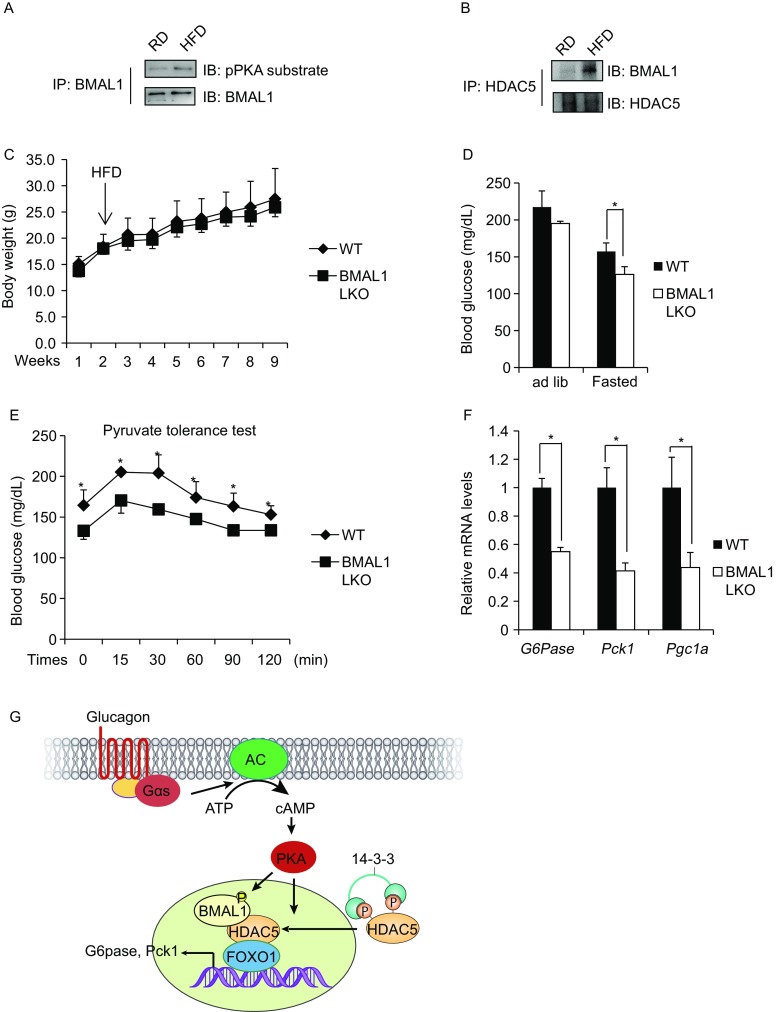
**Deficiency of hepatic BMAL1 attenuates hyperglycemia in HFD-fed mice**. (A) Immunoblot showing phosphorylation levels of BMAL1 in RD- or HFD-fed mice. (B) Immunoblot of BMAL1 recovered from immunoprecipitations of HDAC5 from liver samples in RD- or HFD-fed mice. (C) Weight gain in BMAL1 LKO and control littermates fed on HFD (*n* = 6). (D) Blood glucose levels under ad lib and fasted conditions in BMAL1 LKO and control littermates maintained on HFD (*n* = 6). (E) Pyruvate tolerance test of BMAL1 LKO and control littermates maintained on HFD (*n* = 6). (F) Effect of fasting on mRNA amounts for hepatic gluconeogenic genes in liver of BMAL1 LKO and control littermates maintained on HFD (*n* = 6). (G) Schematic of proposed mechanism. All data are presented as mean ± s.e.m. *P < 0.05

In summary, we have identified BMAL1 as a cAMP-responsive coactivator of HDAC5 to regulate hepatic gluconeogenesis (Fig. 2G). Excessive phosphorylation of BMAL1 might contribute to the hyperglycemia in type 2 diabetes. Dissociation of BMAL1 from HDAC5 in the nucleus is a potential strategy for treating type 2 diabetes associated hyperglycemia.

## Electronic supplementary material

Below is the link to the electronic supplementary material.

Supplementary material 1 (PDF 324 kb)
