# Supplementary material for: BMAL1 functions as a cAMP-responsive coactivator of HDAC5 to regulate hepatic gluconeogenesis
Source: Protein Cell. 2018 Mar 5;9(11):976–80. doi: 10.1007/s13238-018-0514-y (PMC6208480; doi:10.1007/s13238-018-0514-y)
Supplement: Supplementary file 1 — Supplementary material 1 (PDF 324 kb) [file 13238_2018_514_MOESM1_ESM.pdf]

Materials and Methods:

Cells, antibodies, and reagents

Primary hepatocytes were prepared as described (Dentin et al, 2004). Briefly, livers from fed mice were perfused with Hank's balanced salt solution (Invitrogen, Waltham, MA, USA) followed by collagenase (Type IV) (Sigma, St. Louis, MO, USA) at a rate of 6 ml/min through the portal vein. Cells were seeded in medium M199 (Invitrogen, Waltham, MA, USA), supplemented with 0.2% (w/v) bovine serum albumin, 2% (v/v) fetal bovine serum (FBS). The medium was replaced with fresh M199 medium after 2 hours. Cells were then infected with 1 plaque-forming unit per cell (pfu/cell) of Ad-GFP, Ad-BMAL1 or Ad-BMAL1 S42A for 24 h for overexpression and Ad-USi or Ad-BMAL1i for 48 h for RNAi-mediated knockdown. HEK293T cells were maintained in DMEM with 10% FBS and transfected with Lipofectamine 2000 (Invitrogen, Waltham, MA, USA). Anti-pHDAC5 (#3443, AB_2118723), phosphor-PKA substrate (#9621, AB_330304) antibodies were purchased from Cell Signaling Technology (Danvers, MA, USA). Anti-HDAC5 (#ab1439, AB_2118891), Anti-BMAL1 antibody (#ab93806, AB_10675117), Anti-pCREB (Ser133) (#ab32096, AB_731734) and CREB (#ab23515, AB_2292301) antibodies was purchased from Abcam (Cambridge, UK). Forskolin (FSK) and H89 were purchased from Sigma (St. Louis, MO, USA).

Animals and adenovirus

BMAL1 LKO mice were kindly provided by Dr. Yi Liu (Institute for Nutritional Sciences, SIBS, CAS). C57BL/6J mice were purchased from Shanghai Laboratory Animal Center (Shanghai, China). All mice were adapted to colony cages with 12 h light/dark cycle in a temperature-controlled environment with free access to water and standard irradiated rodent diet (5% fat; Research Diet, D12450, New Brunswick, NJ, USA). For high-fat diet (HFD) studies, 4-week-old mice were maintained on HFD (60% fat; Research Diets, D12492) for 8 weeks. For adenovirus injection, 1×10^8 pfu Ad-GFP, Ad-BMAL1 and Ad-BMAL1 S42A were delivered by tail vein injection. For pyruvate tolerance test (PTT), mice were fasted for 16 h beginning at ZT10, and then injected intraperitoneally with sodium pyruvate (1.5 g/kg). Blood was collected from the tail vein at indicated times and glucose levels were measured using a One Touch Ultra Glucometer (Johnson & Johnson, New Brunswick, NJ, USA). Insulin levels were measured by Ultra-Sensitive Mouse Insulin ELISA Kit (Crystal Chem, IL, USA). All animal studies were approved by the Animal Experiment Committee of Tongji University and in accordance with the guidelines of school of medicine, Tongji University. Ad-BMAL1, Ad-BMAL1 S42A and Ad-BMAL1i were kindly provided by Dr. Yi Liu (Institute for Nutritional Sciences, SIBS, CAS). Ad-*G6Pase*-luc, Ad-*G6Pase mCRE*-luc, Ad-*G6Pase mIRE*-luc or Ad-*CRE*-luc adenovirus were kindly provided by Dr. Marc Montminy (Salk Institute, La Jolla, USA)(Liu et al, 2008).

***In vitro* analysis**

Mouse tissues were frozen in liquid nitrogen and kept at -80° C until further use. Livers were homogenized using tissue homogenizer and further sonicated at 4°C in lysis buffer (150 mM NaCl, 50 mM Tris-HCl pH 7.5, 5 mM EDTA, 30 mM sodium pyrophosphate, 30 mM sodium fluoride, 1% Triton-X 100, and protease inhibitor cocktail). Lysates were centrifuged for 10 min at 20,000 x g at 4°C and supernatants were reserved for immunoblot and immunoprecipitation.

Quantitative real-time PCR and immunoblot

Real-time PCR was performed as previously (Lv et al, 2017). Briefly, total RNA was isolated using TRIzol reagent (Invitrogen, Carlsbad, CA, USA) and reverse transcription was done using FastQuant RT kit (Tiangen, Shanghai, China). Real-time PCR was carried out using SuperReal SYBR

Green kit (Tiangen, Shanghai, China) and Lightcycler 96 (Roche, Penzberg, Germany). All reactions were performed in duplicate (Schmittgen & Livak, 2008). Immunoblot and immunoprecipitation were performed as described (Luan et al, 2014).

Luciferase reporter assay

Primary hepatocytes were infected with 1 pfu/cell of Ad-*G6Pase*-luc, Ad-*G6Pase mCRE*-luc, Ad-*G6Pase mIRE*-luc or Ad-*CRE*-luc adenovirus together with Ad-RSV-β-gal adenovirus for 24 h and luciferase assays were performed using Promega GloMax96 system according to the manufacturer's instruction (Luan et al, 2014). β-gal assay was used to normalize the expression levels.

Statistical analysis

All studies were performed on at least three independent occasions. Results are reported as mean±s.e.m. Differences between two groups were assessed using unpaired Student's *t* test. Data involving more than two groups were assessed by analysis of variance (ANOVA) with Bonferroni *post hoc* test. A *p*–value of < 0.05 was considered statistically significant.

Dentin R, Pegorier JP, Benhamed F, Foufelle F, Ferre P, Fauveau V, Magnuson MA, Girard J, Postic C (2004) Hepatic glucokinase is required for the synergistic action of ChREBP and SREBP-1c on glycolytic and lipogenic gene expression. *The Journal of biological chemistry* **279:** 20314-20326

Liu Y, Dentin R, Chen D, Hedrick S, Ravnskjaer K, Schenk S, Milne J, Meyers DJ, Cole P, Yates J, 3rd, Olefsky J, Guarente L, Montminy M (2008) A fasting inducible switch modulates gluconeogenesis via activator/coactivator exchange. *Nature* **456:** 269-273

Luan B, Goodarzi MO, Phillips NG, Guo X, Chen YD, Yao J, Allison M, Rotter JI, Shaw R, Montminy M (2014) Leptin-mediated increases in catecholamine signaling reduce adipose tissue inflammation via activation of macrophage HDAC4. *Cell metabolism* **19:** 1058-1065

Lv S, Li J, Qiu X, Li W, Zhang C, Zhang ZN, Luan B (2017) A negative feedback loop of ICER and NF-kappaB regulates TLR signaling in innate immune responses. *Cell death and differentiation* **24:** 492-499

Schmittgen TD, Livak KJ (2008) Analyzing real-time PCR data by the comparative C(T) method. *Nature protocols* **3:** 1101-1108

Supplemental Figure Legend

Fig. S1. BMAL1 promotes hepatic gluconeogenesis.
(A), Body weight of BMAL1 LKO mice and control littermates under regular diet (n = 6). (B), Blood glucose levels under ad lib and fasted conditions in BMAL1 LKO and control littermates maintained on regular diet (n = 6). (C), Serum insulin levels in BMAL1 LKO and control littermates under fasted conditions. (D), Pyruvate tolerance test of BMAL1 LKO and control littermates maintained on regular diet (n = 6). (E), Effect of fasting on mRNA amounts for hepatic gluconeogenic genes in liver of BMAL1 LKO and control littermates maintained on regular diet (n = 6). (F), BMAL1 mRNA amounts in Ad-GFP or Ad-BMAL1 infected primary hepatocytes (n = 3). (G), BMAL1 mRNA amounts in Ad-USi or Ad-BMAL1i infected primary hepatocytes (n = 3). All data are presented as mean±s.e.m. *P<0.05.

Fig. S2. BMAL1 promotes hepatic gluconeogenesis through HDAC5.
(A), Effect of Ad-BMAL1 on FSK-induced Ad-G6Pase-luc reporter activity in primary hepatocytes (n = 3). (B), Effect of Ad-BMAL1 on FSK-induced Ad-CRE-luc reporter activity in primary hepatocytes (n = 3). (C), Effect of Ad-BMAL1 on FSK-induced Ad-G6Pase-luc, Ad-G6Pase mCRE-luc and Ad-G6Pase mIRE-luc reporter activity in primary hepatocytes (n = 3). (D), Effect of Ad-BMAL1i on FSK-induced Ad-G6Pase-luc reporter activity in primary hepatocytes (n = 3). (E), Effect of Ad-BMAL1 on dephosphorylation of HDAC5 in primary hepatocytes exposed to FSK. (F), Effect of Ad-BMAL1i on dephosphorylation of HDAC5 in primary hepatocytes exposed to FSK. All data are presented as mean±s.e.m. *P<0.05.

Fig. S3. PKA-mediated phosphorylation of BMAL1 promotes its binding to HDAC5.
(A), Colocalization of DsRed-tagged HDAC5 with GFP-tagged BMAL1 in HEK293T cells exposed to FSK. (B), Immunoblot showing effects of FSK treatment on BMAL1 phosphorylation at indicated time in primary hepatocytes. (C), Immunoblot showing phosphorylation levels of BMAL1 in WT mice under ad lib and fasted conditions. (D-E), Immunoblot showing effects of FSK treatment on BMAL1 phosphorylation in HEK293T transfected with indicated constructs. (F), Interaction between Flag-tagged HDAC5 and HA-tagged BMAL1 or BMAL1 KRAA in HEK293T cells. (G), Interaction between Flag-tagged HDAC5 or HDAC5 2SA and HA-tagged BMAL1 in HEK293T cells. (H), Effect of Ad-BMAL1 or Ad-BMAL1 S42A on FSK-induced Ad-*G6Pase*-luc reporter activity in primary hepatocytes (*n* = 3). (I), Effect of Ad-BMAL1 or Ad-BMAL1 S42A on dephosphorylation of HDAC5 in primary hepatocytes exposed to FSK. (J), Blood glucose levels under ad lib and fasted conditions in mice injected with Ad-BMAL1 or Ad-BMAL1 S42A (*n* = 6).

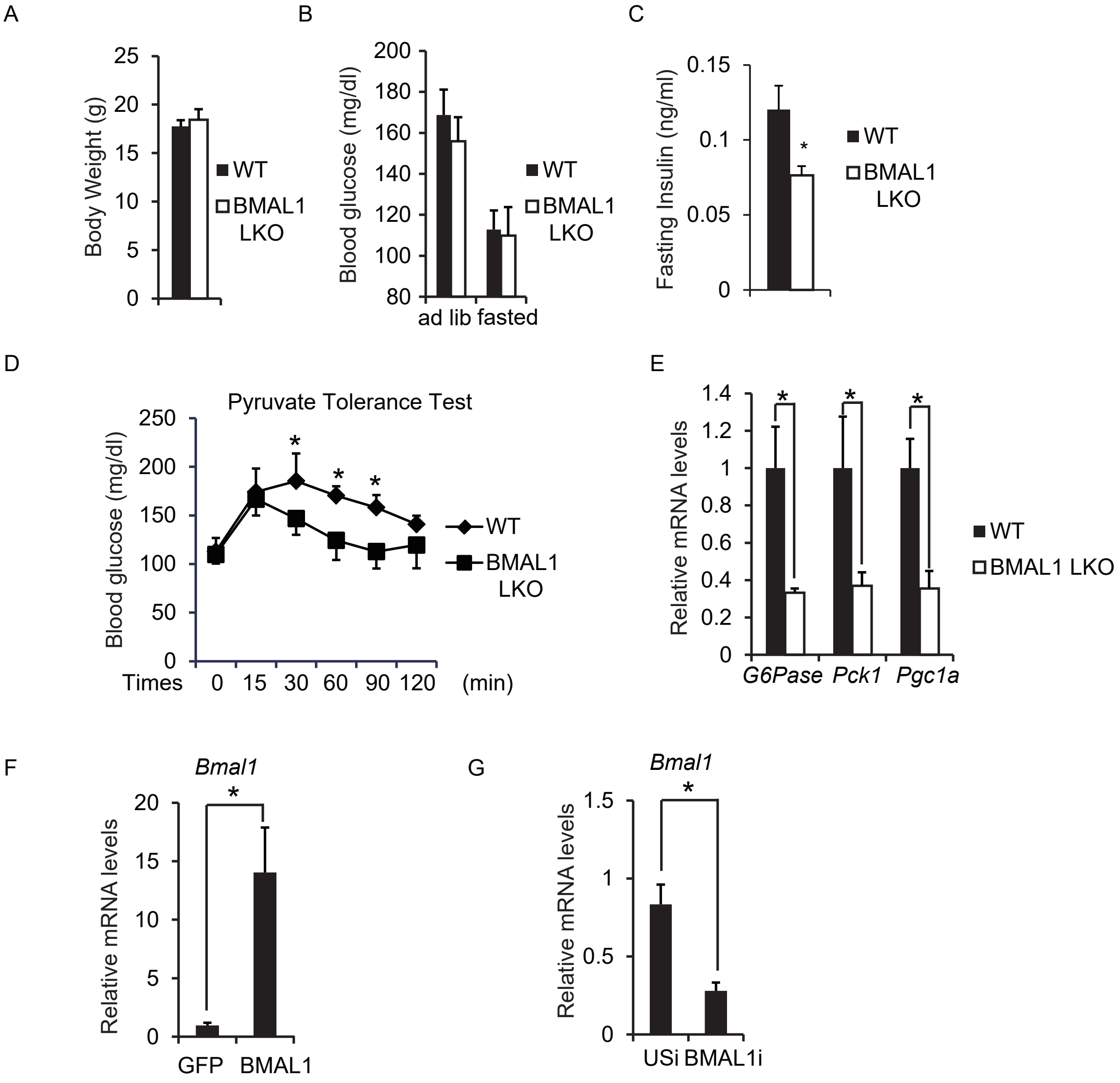
A
Body Weight (g)
25
20
15
10
5
0
WT
BMAL1 LKO
B
Blood glucose (mg/dl)
200
180
160
140
120
100
80
ad lib fasted
WT
BMAL1 LKO
C
Fasting Insulin (ng/ml)
0.15
0.1
0.05
0
*
WT
BMAL1 LKO
D
Pyruvate Tolerance Test
Blood glucose (mg/dl)
250
200
150
100
50
0
*
*
*
Times 0 15 30 60 90 120 (min)
WT
BMAL1 LKO
E
Relative mRNA levels
1.4
1.2
1
0.8
0.6
0.4
0.2
0
*
*
*
G6Pase Pck1 Pgc1a
WT
BMAL1 LKO
F
Bmal1
Relative mRNA levels
20
15
10
5
0
*
GFP BMAL1
G
Bmal1
Relative mRNA levels
1.5
1
0.5
0
*
USi BMAL1i

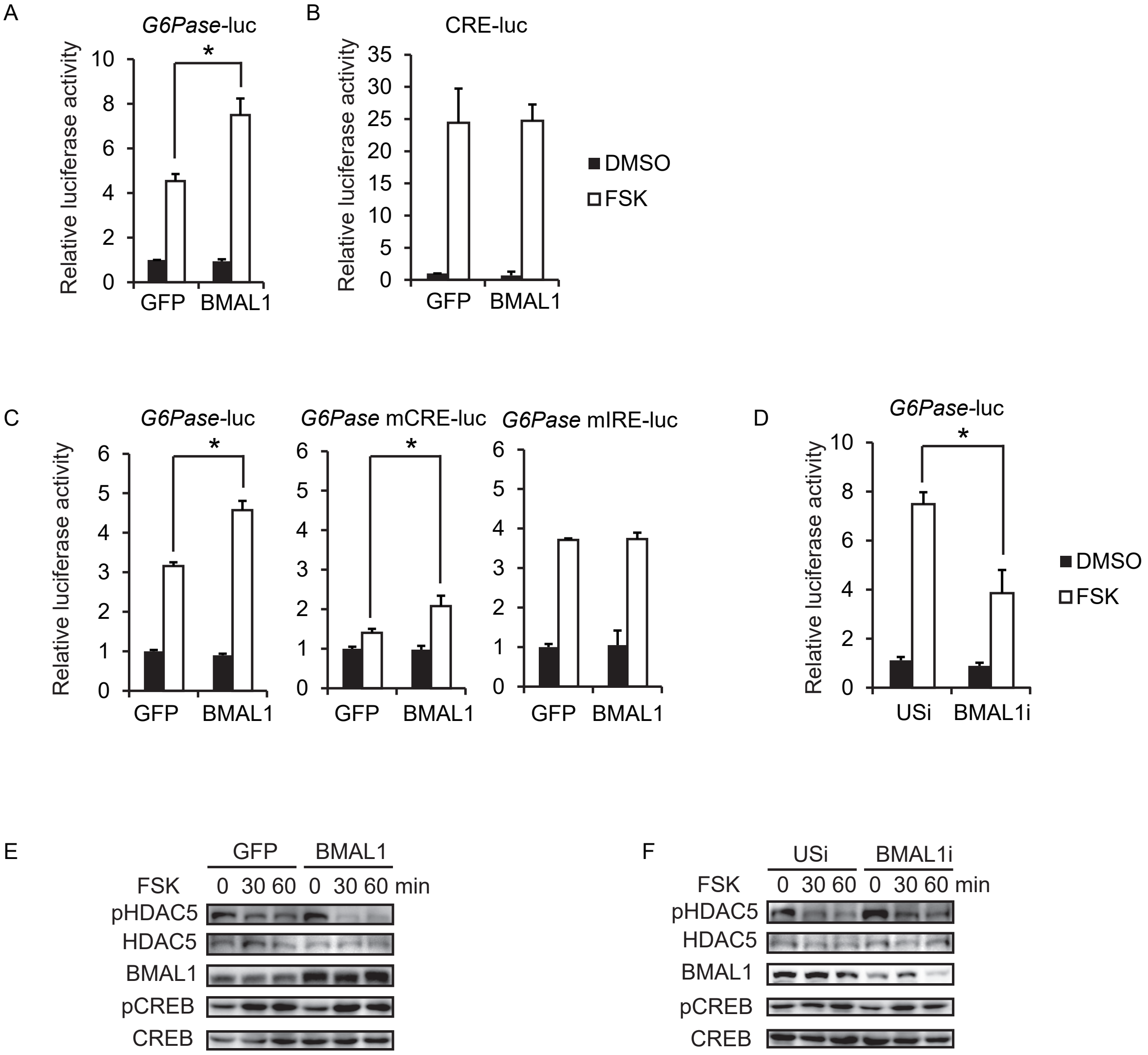
A
G6Pase-luc
Relative luciferase activity
10
8
6
4
2
0
*
GFP
BMAL1
B
CRE-luc
Relative luciferase activity
35
30
25
20
15
10
5
0
GFP
BMAL1
DMSO
FSK
C
G6Pase-luc
G6Pase mCRE-luc
G6Pase mIRE-luc
Relative luciferase activity
6
5
4
3
2
1
0
*
GFP
BMAL1
D
G6Pase-luc
Relative luciferase activity
10
8
6
4
2
0
*
USi
BMAL1i
DMSO
FSK
E
GFP
BMAL1
FSK
0 30 60 0 30 60 min
pHDAC5
HDAC5
BMAL1
pCREB
CREB
F
USi
BMAL1i
FSK
0 30 60 0 30 60 min
pHDAC5
HDAC5
BMAL1
pCREB
CREB

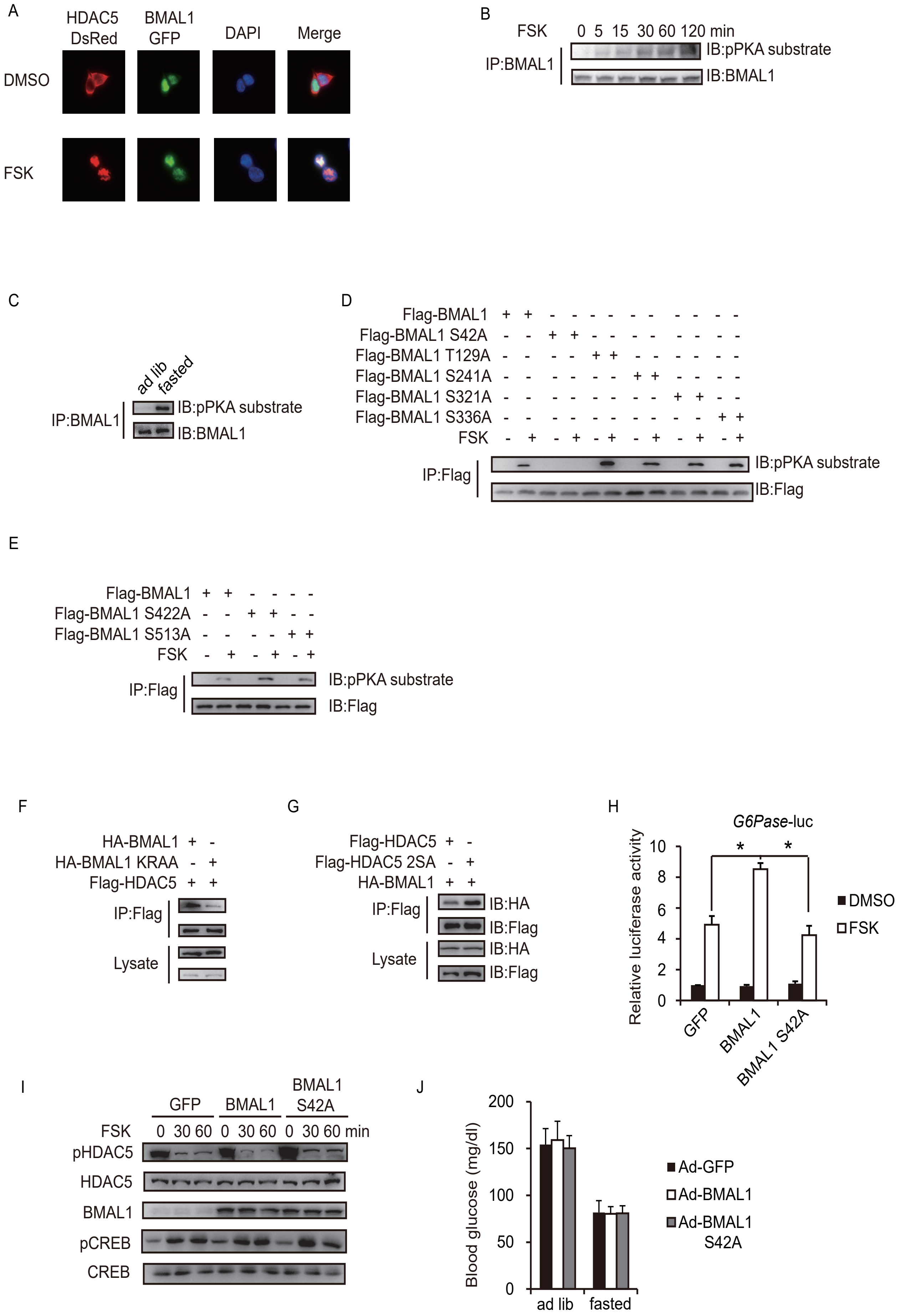
A
HDAC5 DsRed
BMAL1 GFP
DAPI
Merge
DMSO
FSK
B
FSK 0 5 15 30 60 120 min
IP:BMAL1
IB:pPKA substrate
IB:BMAL1
C
ad lib
fasted
IP:BMAL1
IB:pPKA substrate
IB:BMAL1
D
Flag-BMAL1 + + - - - - - - - - - -
Flag-BMAL1 S42A - - + + - - - - - - - -
Flag-BMAL1 T129A - - - - + + - - - - - -
Flag-BMAL1 S241A - - - - - - + + - - - -
Flag-BMAL1 S321A - - - - - - - - + + - -
Flag-BMAL1 S336A - - - - - - - - - - + +
FSK - + - + - + - + - + - +
IP:Flag
IB:pPKA substrate
IB:Flag
E
Flag-BMAL1 + + - - - -
Flag-BMAL1 S422A - - + + - -
Flag-BMAL1 S513A - - - - + +
FSK - + - + - +
IP:Flag
IB:pPKA substrate
IB:Flag
F
HA-BMAL1 + -
HA-BMAL1 KRAA - +
Flag-HDAC5 + +
IP:Flag
Lysate
G
Flag-HDAC5 + -
Flag-HDAC5 2SA - +
HA-BMAL1 + +
IP:Flag
Lysate
IB:HA
IB:Flag
IB:HA
IB:Flag
H
G6Pase-luc
Relative luciferase activity
0
2
4
6
8
10
*
*
GFP
BMAL1
BMAL1 S42A
DMSO
FSK
I
GFP
BMAL1
BMAL1 S42A
FSK 0 30 60 0 30 60 0 30 60 min
pHDAC5
HDAC5
BMAL1
pCREB
CREB
J
Blood glucose (mg/dl)
0
50
100
150
200
ad lib
fasted
Ad-GFP
Ad-BMAL1
Ad-BMAL1 S42A
